# Molecular responses of legumes to abiotic stress: post-translational modifications of proteins and redox signaling

**DOI:** 10.1093/jxb/erab008

**Published:** 2021-01-16

**Authors:** Manuel A Matamoros, Manuel Becana

**Affiliations:** 1 Departamento de Nutrición Vegetal, Estación Experimental de Aula Dei, Consejo Superior de Investigaciones Científicas, Apartado 13034, 50080 Zaragoza, Spain; 2 University of Birmingham, UK

**Keywords:** Abiotic stress, legumes, nitric oxide, nitrogen fixation, post-translational modifications, reactive oxygen/nitrogen/sulfur species, redox signaling, symbiosis

## Abstract

Legumes include several major crops that can fix atmospheric nitrogen in symbiotic root nodules, thus reducing the demand for nitrogen fertilizers and contributing to sustainable agriculture. Global change models predict increases in temperature and extreme weather conditions. This scenario might increase plant exposure to abiotic stresses and negatively affect crop production. Regulation of whole plant physiology and nitrogen fixation in legumes during abiotic stress is complex, and only a few mechanisms have been elucidated. Reactive oxygen species (ROS), reactive nitrogen species (RNS), and reactive sulfur species (RSS) are key players in the acclimation and stress tolerance mechanisms of plants. However, the specific redox-dependent signaling pathways are far from understood. One mechanism by which ROS, RNS, and RSS fulfil their signaling role is the post-translational modification (PTM) of proteins. Redox-based PTMs occur in the cysteine thiol group (oxidation, *S*-nitrosylation, *S*-glutathionylation, persulfidation), and also in methionine (oxidation), tyrosine (nitration), and lysine and arginine (carbonylation/glycation) residues. Unraveling PTM patterns under different types of stress and establishing the functional implications may give insight into the underlying mechanisms by which the plant and nodule respond to adverse conditions. Here, we review current knowledge on redox-based PTMs and their possible consequences in legume and nodule biology.

## Teaser

Redox signaling and protein post-translational modifications of proteins are important mechanisms of perception response to abiotic stress in legumes.

## Introduction

**Fig. 1.**
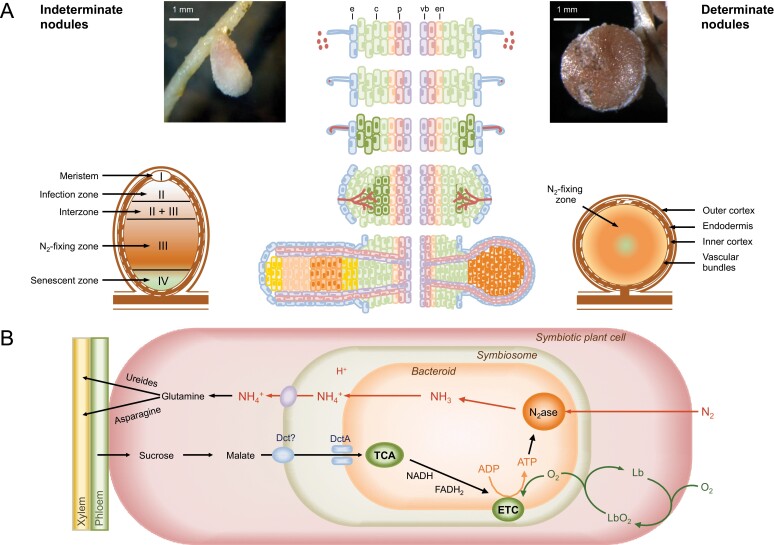
Scheme showing the infection process, the major differences between indeterminate and determinate nodules, and some key metabolic pathways in the nodule cells. (A) Briefly, the infection process is as follows: legume roots release flavonoids to the rhizosphere which induce the production of nodulation (Nod) factors in compatible rhizobia (depicted as red dots next to root hairs); Nod factors are recognized by root receptors that activate the symbiotic signaling pathway; rhizobia enter the root through hairs that curve and trap the bacteria inside a curl; invaginations of the cell membrane form infection threads that permit the invasion of the root cortex by rhizobia; a new nodule meristem forms underneath the site of infection; as the nodule grows, the bacteria are released into membrane-bound compartments, the symbiosomes, inside the nodule cells, where the bacteria differentiate into bacteroids and start N_2_ fixation. As a result, two major types of nodules are formed. Indeterminate nodules of *Medicago truncatula* and crops such as pea and alfalfa contain a persistent meristem and are generally elongated with a longitudinal gradient of age. Four zones can be distinguished from the apex (distal) to the base (proximal) regions: zone I (meristem), zone II (infection), zone III (N_2_-fixing), and zone IV (senescent). Determinate nodules of *Lotus japonicus* and of crops such as soybean and common bean lack permanent meristems and are usually spherical. In this case, N_2_ fixation takes place in the central infected zone, which also contains uninfected or interstitial cells. Abbreviations of cell layers: c, cortex; e, epidermis; en, endodermis; p, pericycle; vb, vascular bundle. (B) Some key processes in the symbiotic nodule cells. Sucrose from the shoot is metabolized to malate that is transported into bacteroids through dicarboxylate transporters (Dct). In the bacteroids, malate is oxidized, providing energy through the tricarboxylic acid (TCA) cycle and the electron transport chain (ETC) for nitrogenase (N_2_ase) activity. Fixed nitrogen in the form of ammonium is transported back to the plant where, with some exceptions, it is assimilated into the amides glutamine and asparagine in indeterminate nodules or ureides in determinate nodules. In the cytoplasm of infected cells, leghemoglobin (Lb) transports and delivers O_2_ to the symbiosomes at a low steady concentration to avoid the inhibition of nitrogenase, but to simultaneously allow high rates of bacteroid respiration. After Oldroyd *et al.,* (2011), Popp and Ott (2011), and Hichri *et al.,* (2015).

Crops provide the vast majority of global food requirements. However, modern agriculture relies greatly on the supply of fertilizers, especially nitrogen, and the demand is expected to rise due to population growth. Industrially, nitrogen is reduced to ammonia by the Haber-Bosch reaction, yet this process has negative consequences for the environment because it depends on fossil fuel consumption. Also, the use of excess fertilizers in the field often leads to water pollution, causing problems such as eutrophication or poisoning of drinking water (Erisman *et al.*, 2011). Legumes are the third largest family of angiosperms and the second most important crop worldwide. They have the capacity to fix atmospheric N_2_ in root nodules, which are formed after infection of host plants by rhizobia present in the soil. Further details on the rhizobia-legume symbiosis and on the structure of legume nodules can be found in the reviews by Oldroyd (2013) and Roy *et al.* (2020), and in Fig. 1. Bacterial nitrogenase activity injects around 40 million tons of nitrogen into agricultural systems every year, thus reducing the demand for nitrogen fertilizers and contributing to sustainable agriculture (Udvardi and Poole, 2013; Gresshoff *et al.*, 2015).

Mankind is confronted with environmental challenges that are predicted to worsen in the upcoming decades. Global change models predict higher temperatures and extreme weather conditions (Schmidhuber and Tubiello, 2007). This scenario might increase plant exposure to abiotic stresses and negatively affect crop production. In legumes, symbiotic nitrogen fixation (SNF) is particularly sensitive to adverse conditions, including drought (Ladrera *et al.*, 2007; Naya *et al.*, 2007; Aranjuelo *et al.*, 2014; Nasr Esfahani *et al.*, 2014; Dhanushkodi *et al.*, 2018), salinity (Jebara *et al.*, 2005; López *et al.*, 2008), and heavy metals (Marino *et al.*, 2013; Baig *et al.*, 2018). Plants under stress conditions produce enhanced amounts of reactive oxygen species (ROS), reactive nitrogen species (RNS), and reactive sulfur species (RSS) that need to be kept under control by antioxidant enzymes and metabolites, to avoid toxicity and allow their participation in signaling events (Becana *et al.*, 2010; Mittler, 2017; Begara-Morales *et al.*, 2019; Hancock, 2019). Hydrogen peroxide (H_2_O_2_), superoxide radical (O_2_^.-^), nitric oxide (NO), and hydrogen sulfide (H_2_S) are key players in the acclimation of plants to abiotic stress (Choudhury *et al.*, 2017; Hancock, 2019). A substantial part of the signaling capacity and bioactivity of ROS, RNS, and RSS stems from their ability to cause post-translational modifications (PTMs), both reversible and irreversible, of amino acid side chains of proteins. PTMs may trigger changes in protein function, leading to increases or decreases in activity and, in certain cases, protein degradation (Friso and van Wijk, 2015; Gupta *et al.*, 2020). Here, we review current knowledge on redox-based PTMs of proteins in legumes. PTM-driven changes in the function of transcription factors and enzymes may be connected to alterations in gene expression, physiology, and metabolism that allow plant acclimation and tolerance to adverse environmental conditions. A deep understanding of how plant proteins are regulated by alterations in the redox state through PTMs can be of extraordinary significance in biotechnology and breeding programs.

### Production of ROS, RNS, and RSS in legumes during abiotic stress

Plants perceive changes in environmental conditions through the activation of channels and/or sensors and the initiation of signaling cascades, which entail alterations in calcium and other ion fluxes, shifts in hormonal balance, protein phosphorylation, and ROS- and RNS-induced PTMs (Choudhury *et al.*, 2017; Kollist *et al.*, 2019; Lamers *et al.*, 2020). In the leaves, ROS are mostly produced in the apoplast by NADPH oxidases [also known as Respiratory Burst Oxidase Homologues (RBOHs)] and by other oxidases and peroxidases, but also in the chloroplasts, mitochondria, and peroxisomes (Choudhury *et al.*, 2017). Very recently, a sensor that detects H_2_O_2_ at the cell surface has been reported. This important discovery emphasizes the key role of ROS in the perception and response of plant cells to environmental stress (Wu *et al.*, 2020). As for NO, despite intensive research its production in plants is only partially understood. The best described enzymatic sources are cytosolic nitrate reductase, the mitochondrial electron transport chain, and the oxidation of aminated molecules (Astier *et al.*, 2018; Umbreen *et al.*, 2018). In plant cells, H_2_S is produced in the plastids by sulfite reductase as an intermediate of assimilatory sulfate reduction, and then incorporated into *O*-acetylserine by *O*-acetylserine thiol lyase (OASTL) to form cysteine (Cys) in the plastids, cytosol, and mitochondria. However, OASTL can also catalyse the reverse reaction, generating H_2_S from Cys. In the cytoplasm, the main source of H_2_S is l-Cys desulfhydrase, which decomposes Cys to H_2_S, ammonia, and pyruvate. In the mitochondria, cyanoalanine synthase catalyses the reaction between Cys and cyanide to produce β-cyanoalanine and H_2_S (Aroca *et al.*, 2018).

The stress-induced senescence of legume nodules is characterized by a rapid decrease in SNF caused by the decline of leghemoglobin (Lb) content and the reduction of key activities such as sucrose synthase, nitrogenase, and nitrogen assimilatory enzymes (Aranjuelo *et al.*, 2014). In nodules, ROS are generated by NADPH oxidase activity in plant membranes, the oxidative metabolism of peroxisomes, the respiration of mitochondria, the autoxidation of oxyferrous Lb in the cytoplasm, and the oxidation of nitrogenase, ferredoxin, and hydrogenase in the bacteroids (Becana and Klucas, 1992; Santos *et al.*, 2001; Marino *et al.*, 2012; Puppo *et al.*, 2013; Arthikala *et al.*, 2017). Likewise, NO can be formed in nodules through the bacterial denitrification pathway, the reduction of NO_3_^-^ by plant nitrate reductase, and the electron transport chain of mitochondria in the hypoxic tissue (Meakin *et al.*, 2007; Horchani *et al.*, 2011; Calvo-Begueria *et al.*, 2018). It has been recently reported that H_2_S promotes SNF (Zou *et al.*, 2019). Although the synthesis of H_2_S in nodules has not been investigated in detail yet, it is probably generated by both plant and bacterial enzymes. A recent study showed that deletion in *Sinorhizobium (Ensifer) fredii* of cystathionine γ-lyase, an enzyme that participates in the production of H_2_S in some bacteria and mammals, caused a sharp decrease in H_2_S content, inhibition of nitrogenase activity, and accumulation of H_2_O_2_ and malondialdehyde in soybean (*Glycine max*) nodules (Zou *et al.*, 2020).

Stress-induced alterations of ROS, RNS, and RSS amounts may also modify the ratios of the redox couples ascorbate/dehydroascorbate and reduced glutathione/glutathione disulfide, as well as the expression and activity of antioxidant enzymes, in the leaves (Corpas *et al.*, 2008; Noreen and Ashraf, 2009; Rodríguez-Serrano *et al.*, 2009; Rubio *et al.*, 2009; Navascués *et al.*, 2012b; Hancock, 2019) and nodules (Marino *et al.*, 2007; Naya *et al.*, 2007; Nasr Esfahani *et al.*, 2014; Marquez-Garcia *et al.*, 2015). In general, stress-induced redox changes in cells and organelles entail an oxidative shift. However, the mechanisms by which variations in the cellular redox state are transduced into specific responses are unclear. Important factors are the concentration, exposure time, subcellular location, and type of ROS, RNS, and RSS. In each cell compartment, a consequence of the loss of homeostasis of these reactive molecules may be the modification of susceptible amino acids. These are mostly the sulfur-containing amino acids Cys and methionine (Met), but also tyrosine (Tyr), lysine (Lys), and arginine (Arg). These changes may potentially alter the activity of enzymes and transcription factors, thus regulating key metabolic processes, signaling pathways, and gene expression.

### Proteomics provides insight into the mechanisms of stress adaptation of legumes

Over the last 15 years, the field of proteomics has revolutionized the identification and quantification of plant proteins involved in the responses to abiotic stress (Kosova *et al*., 2018). These studies are invaluable as they provide a global perspective of plant adaptation to adverse conditions. Nevertheless, despite the extensive inventory of plant proteins involved in the stress response, many issues still need to be examined in detail. These include the functional characterization of proteins, the assessment of the effects of PTMs on the activity, location and interaction with other proteins, and the susceptibility of modified proteins to degradation under specific environmental conditions.

In legumes, a considerable number of proteomic studies have been carried out to unravel the mechanisms of stress tolerance (reviewed by Rathi *et al.* 2016). For example, comparison of dehydration-sensitive and dehydration-tolerant cultivars of chickpea (*Cicer arietinum*) revealed differential expression of many proteins. Tolerance was attributed, at least in part, to altered expression of proteins involved in ROS catabolism (Subba *et al.*, 2013). In the same species, up-regulation of expression of proteins with chaperone-like functions and of proteins involved in ROS homeostasis was associated with improved germination and early seedling growth under sub-optimal soil–water conditions (Vessal *et al.*, 2012). Other studies also suggested an important role of ROS in stress acclimation. Based on the proteomics of salt-tolerant and salt-sensitive genotypes of soybean, it was concluded that the tolerant genotype possessed a higher capacity to secure energy supply and to maintain ROS homeostasis, photosynthetic rate, and ethylene synthesis (Ma *et al.*, 2012). Salinity-induced changes in the root proteome of pea (*Pisum sativum*) pointed to the possible existence of a signal transduction pathway involving H_2_O_2_ and the antioxidant enzyme superoxide dismutase (Kav *et al.*, 2004). Overall, these studies highlight the importance of ROS homeostasis in stress tolerance.

The proteomic studies of nodules lag well behind those of leaves. Investigations in the model legumes *Lotus japonicus* and *Medicago truncatula* have provided insight into quantitative expression of proteins in plant organs, PTMs, and the mechanisms that regulate the symbiosis. The comparative analysis of *L. japonicus* root and nodule proteomes suggests that, in general, nodule proteins contain higher amounts of PTMs (Dam *et al.*, 2014). This might be related to a better capacity to face, and adapt to, stress conditions of metabolically active nodules, compared with the less active roots. Furthermore, to our knowledge, in the largest study to date on legume proteomics, >23 000 proteins, 20 000 phosphorylation sites, and 700 Lys acetylation sites were identified in *M. truncatula* and its symbiont *S. meliloti* (Marx *et al.*, 2016). This study established a core of *M. truncatula* proteins expressed in all organs, identified a subset of proteins that displayed organ-specific regulation, and assigned putative functions to several uncharacterized proteins. Compared with roots, significant regulatory and phosphorylation events were identified during nodule development in proteins involved in oxygen transport, immune response, and senescence. This extensive resource might be useful for future studies aimed at identifying proteins and PTMs that participate in the stress response. Quantitative proteomics in *M. truncatula* revealed major changes in nodule proteins under drought conditions. This stress caused a decrease in the amount of proteins involved in SNF and carbon metabolism in the bacteroids, as well as of sucrose synthase and enzymes of the Met and ethylene biosynthetic pathways in the nodule host cells (Larrainzar *et al*., 2009; 2014). Another study found that exposure of soybean nodules to toxic heavy metals, even at low concentrations, increased the expression of proteins involved in development, hormone signaling, and stress responses (Baig *et al.*, 2018).

### Post-translational modifications of proteins and redox signaling under abiotic stress

Many proteins undergo one or more PTMs throughout their lifetime. These can be reversible, such as the formation of disulfide bonds, methionine sulfoxides (MetSO), *S*-nitrosylation, and *S*-glutathionylation, or irreversible, such as carbonylation and glycation (Friso and van Wijk, 2015). Redox modifications of proteins may affect their activity, stability, location, and interaction with other proteins. Protein PTMs constitute a fast and versatile mechanism by which plants can respond to the frequent environmental constraints faced in natural and agricultural systems. In the next sections, current knowledge of the role of redox-based protein PTMs in the response of legumes to abiotic stress is reviewed.

### Methionine sulfoxidation

**Fig. 2.**
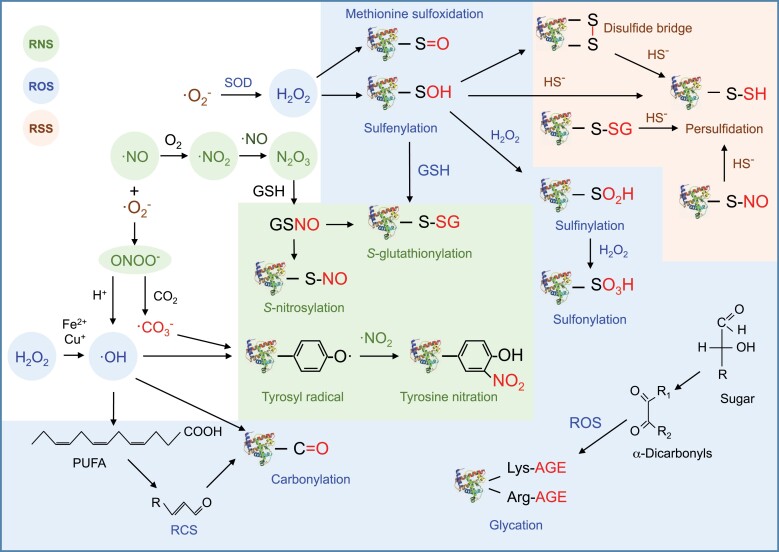
Redox-dependent PTMs. Met residues can be oxidized by hydrogen peroxide (H_2_O_2_) to Met sulfoxides. The *S* and *R* stereoisomers are specifically reduced back to Met by methionine sulfoxide reductases A and B, respectively. Oxidation of deprotonated thiols of Cys residues (–S^-^) by H_2_O_2_ leads to the formation of sulfenic acid (–SOH), which may react with another thiol to form disulfides (–S–S–). This modification can be reverted by thioredoxins and glutaredoxins. The –SOH group can be an intermediate to other redox modifications (see below) or be further oxidized to sulfinic acid (–SO_2_H) and sulfonic acid (–SO_3_H). *S*-nitrosylation (–SNO) is mostly mediated by nitrogen oxides and *trans*-nitrosylating agents such as *S*-nitrosoglutathione (GSNO). *S*-glutathionylation (–SSG) occurs by two main mechanisms: reaction of the target protein with GSNO, and reaction of reduced glutathione (GSH) with –SOH. The reaction of hydrogen sulfide (HS^-^) with –SOH, –SNO, –SSG, or disulfide bridges induces persulfidation (–SSH). Peroxynitrite (ONOO^-^) is formed by the reaction of nitric oxide (NO) with superoxide (O_2_^.-^) radicals. In turn, radicals derived from ONOO^-^ breakdown oxidize Tyr residues to tyrosyl radicals; these react with nitrogen dioxide (NO_2_), produced from ONOO^-^ decomposition, to yield NO_2_–Tyr. The direct oxidation of Lys, Arg, Pro, and Thr by hydroxyl radicals (·OH) incorporates the carbonyl moiety into proteins. Alternatively, oxidation of a polyunsaturated fatty acid (PUFA; a simplified representation is shown lacking part of the aliphatic chain) produces unstable lipid hydroperoxides that decompose to secondary products known as reactive carbonyl species (RCSs). These react with amino acid side chains and generate carbonyl derivatives. Moreover, Arg and Lys residues may react with reducing sugars or α-dicarbonyls such as glyoxal and methylglyoxal, generating glycation products that are readily oxidized to form relatively stable advanced glycation end products (AGEs).

Under stress conditions, the redox state of the apoplast, cell cytoplasm and/or organelles may shift to a more oxidizing state and induce the oxidation of certain Met residues of proteins to a mixture of Met-*S*-sulfoxide and Met-*R*-sulfoxide (Fig. 2). To reverse this oxidation, most organisms, including plants, have evolved two monomeric methionine sulfoxide reductases (MsrA and MsrB) that reduce the *S* and *R* epimers, respectively (Tarrago *et al.*, 2009). Met oxidation may alter protein structure and function, making this modification of regulatory significance in redox signaling. In vertebrates, oxidation of Met residues of calmodulin disrupts downstream calcium-mediated signaling and targets the protein for proteasomal degradation (Snijder *et al.*, 2011). In *Arabidopsis thaliana*, the *in vivo* oxidation of Met-538 of nitrate reductase inhibits phosphorylation of serine-534 (Hardin *et al.*, 2009). Other studies indicate that aging is associated with the accumulation of oxidized Met in proteins, which increases their susceptibility to degradation by the proteasome (Stadtman *et al.*, 2005). In some cases, however, Met oxidation does not seem to affect protein function, and it has been hypothesized that Met residues could function as ultimate endogenous antioxidants in proteins, providing effective scavenging of oxidants before they can attack residues that are critical for structure or function (Stadtman *et al.*, 2005).

**Fig. 3.**
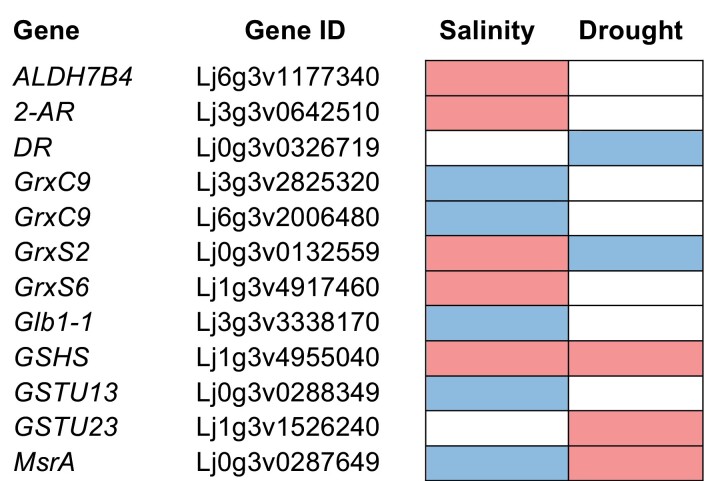
Expression profile of genes involved in redox homeostasis in the shoots of *Lotus japonicus* plants exposed to drought or salt stress. Gene up-regulation (>two-fold) and down-regulation (<0.5-fold) are indicated in red and blue, respectively. Gene IDs are given according to the *L. japonicus* MG-20 genome v3.0 and data were retrieved from the *L. japonicus* Expression Atlas (Lotus Base; https://lotus.au.dk). Transcriptomic data under salt and drought stress were published, respectively, by Sanchez *et al.,* (2008) and Díaz *et al.,* (2010). Abbreviations: ALDH, aldehyde dehydrogenase; AR, alkenal reductase; DR, dehydroascorbate reductase; Glb, phytoglobin; Grx, glutaredoxin; GSHS, glutathione synthetase; GSTU, glutathione transferase tau family; Msr, methionine sulfoxide reductase.

**Fig. 4.**
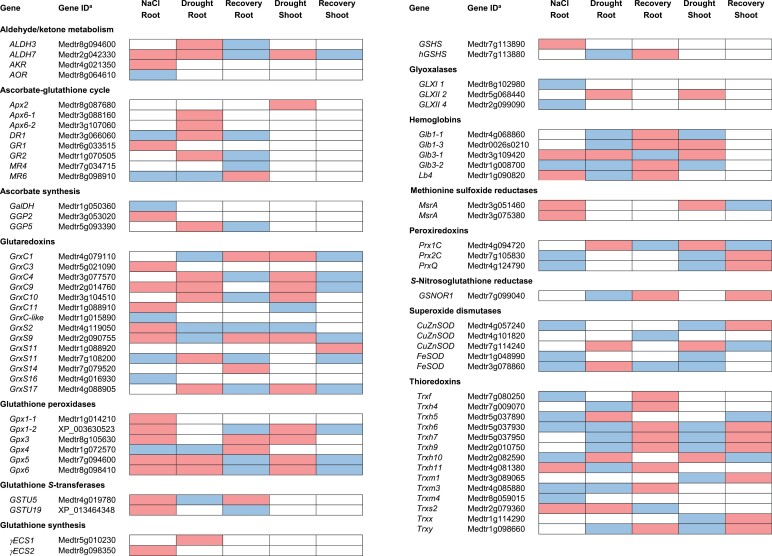
Expression profile of genes involved in redox homeostasis in the roots and shoots of *Medicago truncatula* plants exposed to drought or salt stress. Gene up-regulation (>two-fold) and down-regulation (<0.5-fold) are indicated in red and blue, respectively. Gene IDs are given according to the *M. truncatula* genome v4.0. Transcriptomic data of roots and shoots under drought stress were retrieved from the Gene Expression Atlas (https://mtgea.noble.org/v3/) and those of salt stress in the roots were published by Li *et al*., (2009). Gene sequences were obtained from Legume IP (http://plantgrn.noble.org/LegumeIP/gdp/), except for *Gpx1-2* and *GSTU19*, which were obtained from GenBank. The probes used to determine gene expression profiles are listed in Table 1. Abbreviations: AKR, aldo-keto reductase; ALDH, aldehyde dehydrogenase; Apx, ascorbate peroxidase; AOR, alkenal/one oxidoreductase; DR, dehydroascorbate reductase; γECS, γ-glutamylcysteine synthetase; GalDH, L-galactono-1,4-lactone dehydrogenase; GGP, GDP-L-galactose phosphorylase; Glb, phytoglobin; GLX, glyoxalase; Gpx, glutathione peroxidase; GR, glutathione reductase; Grx, glutaredoxin; GSHS, glutathione synthetase; GSNOR, *S*-nitrosoglutathione reductase; GSTU, glutathione *S*-transferase tau family; hGSHS, homoglutathione synthetase; Lb, leghemoglobin; MR, monodehydroascorbate reductase; Msr, methionine sulfoxide reductase; Prx, peroxiredoxin; SOD, superoxide dismutase; Trx, thioredoxin.

Little is known about Met sulfoxidation in legumes. Genome-wide datasets of the two model legumes available at the Lotus Base and *M. truncatula* Gene Expression Atlas (He *et al.*, 2009; Mun *et al.*, 2016) reveal the expression of four *MsrA* and three *MsrB* genes in *L. japonicus,* and of four *MsrA* and five *MsrB* genes in *M. truncatula*. All genes are expressed in nodules, albeit some of them in very low levels. Transcriptional induction in response to drought and/or salt stress of one *MsrA* gene in *L. japonicus* shoots (Fig. 3; Díaz *et al.*, 2010), and of two *MsrA* genes in *M. truncatula* roots and/or shoots (Fig. 4; Table 1; Li *et al.*, 2009), suggests a role of Msr enzymes in the regulation of Met redox homeostasis and in the tolerance to abiotic stress.

**Table 1.** Affimetrix *M. truncatula* Gene Chip probesets used for the gene expression analysis

Gene	Gene ID	Probeset ID	Gene	Gene ID	Probeset ID
Aldehyde/ketone metabolism			Glutathione synthesis		
*ALDH3*	Medtr8g094600	Mtr.9402.1.S1_at	*γECS1*	Medtr5g010230	Mtr.5743.1.S1_at
*ALDH7*	Medtr2g042330	Mtr.9030.1.S1_at	*γECS2*	Medtr8g098350	Mtr.26628.1.S1_s_at
*AKR*	Medtr4g021350	Mtr.46053.1.S1_at	*GSHS*	Medtr7g113890	Mtr.40809.1.S1_at
*AOR*	Medtr8g064610	Mtr.44591.1.S1_at	*hGSHS*	Medtr7g113880	Mtr.37763.1.S1_at
Ascorbate-glutathione cycle			Glyoxalases		
*Apx2*	Medtr8g087680	Mtr.6533.1.S1_at	*GLXI1*	Medtr8g102980	Mtr.43350.1.S1_at
*Apx6-1*	Medtr3g088160	Mtr.12244.1.S1_s_at	*GLXII2*	Medtr5g068440	Mtr.43695.1.S1_at
*Apx6-2*	Medtr3g107060	Mtr.2925.1.S1_at	*GLXII4*	Medtr2g099090	Mtr.38317.1.S1_at
*DR1*	Medtr3g066060	Mtr.40092.1.S1_at	Hemoglobins		
*GR1*	Medtr6g033515	Mtr.10610.1.S1_at	*Glb1-1*	Medtr4g068860	Msa.878.1.S1_at
*GR2*	Medtr1g070505	Mtr.38809.1.S1_at	*Glb1-3*	Medtr0026s0210	Mtr.29072.1.S1_s_at
*MR4*	Medtr7g034715	Mtr.38020.1.S1_at	*Glb3-1*	Medtr3g109420	Mtr.47990.1.S1_at
*MR6*	Medtr8g098910	Mtr.44027.1.S1_at	*Glb3-2*	Medtr1g008700	Mtr.10341.1.S1_at
Ascorbate synthesis			*Lb4*	Medtr1g090820	Mtr.8550.1.S1_s_at
*GalDH*	Medtr1g050360	Mtr.11321.1.S1_at	Methionine sulfoxide reductases		
*GGP2*	Medtr3g053020	Mtr.37321.1.S1_at	*MsrA*	Medtr3g051460	Mtr.11716.1.S1_at
*GGP5*	Medtr5g093390	Mtr.37322.1.S1_at	*MsrA*	Medtr3g075380	Mtr.11204.1.S1_at
Glutaredoxins			Peroxiredoxins		
*GrxC1*	Medtr4g079110	Mtr.41411.1.S1_at	*Prx1C*	Medtr4g094720	Mtr.11099.1.S1_at
*GrxC3*	Medtr5g021090	Mtr.40741.1.S1_at	*Prx2C*	Medtr7g105830	Mtr.25633.1.S1_at
*GrxC4*	Medtr3g077570	Mtr.50976.1.S1_at	*PrxQ*	Medtr4g124790	Mtr.10844.1.S1_at
*GrxC9*	Medtr2g014760	Mtr.44436.1.S1_at	*S*-Nitrosoglutathione reductase		
*GrxC10*	Medtr3g104510	Mtr.22341.1.S1_at	*GSNOR1*	Medtr7g099040	Mtr.21158.1.S1_at
*GrxC11*	Medtr1g088910	Mtr.42692.1.S1_at	Superoxide dismutases		
*GrxC-like*	Medtr1g015890	Mtr.24815.1.S1_at	*CuZnSOD*	Medtr4g057240	Mtr.15585.1.S1_at
*GrxS2*	Medtr4g119050	Mtr.34638.1.S1_at	*CuZnSOD*	Medtr4g101820	Mtr.17367.1.S1_at
*GrxS9*	Medtr2g090755	Mtr.35755.1.S1_at	*CuZnSOD*	Medtr7g114240	Mtr.16601.1.S1_at
*GrxS11*	Medtr1g088920	Mtr.32795.1.S1_x_at	*FeSOD*	Medtr1g048990	Mtr.37849.1.S1_at
*GrxS11*	Medtr7g108200	Mtr.34408.1.S1_s_at	*FeSOD*	Medtr3g078860	Mtr.42746.1.S1_at
*GrxS14*	Medtr7g079520	Mtr.14770.1.S1_at	Thioredoxins		
*GrxS16*	Medtr4g016930	Mtr.19479.1.S1_at	*Trxf*	Medtr7g080250	Mtr.43208.1.S1_at
*GrxS17*	Medtr4g088905	Mtr.12551.1.S1_at	*Trxh4*	Medtr7g009070	Mtr.26137.1.S1_at
Glutathione peroxidases			*Trxh5*	Medtr5g037890	Mtr.9873.1.S1_at
*Gpx1-1*	Medtr1g014210	Mtr.48948.1.S1_at	*Trxh6*	Medtr5g037930	Mtr.12857.1.S1_at
*Gpx1-2*	XP_003630523	Mtr.12331.1.S1_at	*Trxh7*	Medtr5g037950	Mtr.9679.1.S1_at
*Gpx3*	Medtr8g105630	Msa.2641.1.S1_at	*Trxh9*	Medtr2g010750	Mtr.5202.1.S1_s_at
*Gpx4*	Medtr1g072570	Mtr.48820.1.S1_at	*Trxh10*	Medtr2g082590	Mtr.38559.1.S1_at
*Gpx5*	Medtr7g094600	Mtr.44501.1.S1_at	*Trxh11*	Medtr4g081380	Mtr.18521.1.S1_at
*Gpx6*	Medtr8g098410	Msa.1887.1.S1_at	*Trxm1*	Medtr3g089065	Mtr.42854.1.S1_at
Glutathione *S*-transferases			*Trxm3*	Medtr4g085880	Mtr.43824.1.S1_at
*GSTU5*	Medtr4g019780	Mtr.40588.1.S1_at	*Trxm4*	Medtr8g059015	Mtr.13972.1.S1_at
*GSTU19*	XP_013464348	Mtr.12287.1.S1_at	*Trxs2*	Medtr2g079360	Mtr.40666.1.S1_at
			*Trxx*	Medtr1g114290	Mtr.12884.1.S1_at
			*Trxy*	Medtr1g098660	Mtr.15318.1.S1_at

### Sulfenylation

Together with Met, Cys is a principal target for redox-dependent PTMs in cells. This is due to the versatile chemistry of the sulfur atom that can display oxidation states ranging from –2 to +6, on account of the availability of empty *d*-orbitals for bonding. The sulfur of Cys is fully reduced but, because of its low redox potential, the thiol side chain of proteins can readily undergo a range of oxidative PTMs (Fig. 2). However, not all Cys residues are equally amenable to modification. Their reactivity depends on the residue accessibility, protein microenvironment, and pK_a_ value. Usually, only thiols with low pK_a_ play key roles in catalysis and serve as important sites for PTMs (Go *et al.*, 2015). Thus, deprotonated thiolates (R-S^-^) are reversibly oxidized by ROS to form sulfenic acids (R-SOH) and disulfide bridges (R-S-S-R’). Further oxidation of the former may lead to the formation of sulfinic (R-SO_2_H) and sulfonic (R-SO_3_H) acids (Fig. 2). Sulfonic acid formation seems to be irreversible but the other modifications can be reversed by thioredoxins (Trxs), glutaredoxins (Grxs), and sulfiredoxins (Meyer *et al.*, 2009; Sevilla *et al.*, 2015).

The identification of sulfenylated plant proteins, although challenging due to the instability of sulfenic acids, can be useful to discover redox sensors and as yet unknown components of ROS-mediated signaling cascades. It can also help establish the role of this PTM in the regulation of plant cell metabolism. Very recently, using a state-of-the-art chemoproteomics approach, Huang *et al.* (2019) mapped sulfenylated sites on more than 1000 *A. thaliana* proteins. Remarkably, the first extracellular sensor of H_2_O_2_, Hydrogen-Peroxide induced Calcium increases 1 (HPCA1), characterized in *A. thaliana* is composed of an intracellular kinase domain and an apoplastic extracellular domain. The latter has two pairs of Cys residues that are oxidized by H_2_O_2_ to form R-SOH and disulfide bonds. These PTMs cause a conformational change that triggers intracellular kinase activity and calcium influx into the cell, thus activating signaling pathways (Wu *et al.*, 2020). In *M. truncatula*, the use of chemical and genetic probes combined with mass spectrometry analyses allowed the identification of sulfenylated proteins in inoculated roots and mature nodules (Oger *et al.*, 2012). During the onset of symbiosis, most sulfenylated root proteins are involved in redox signaling and defense, whereas enzymes involved in carbohydrate and amino acid metabolism are the predominant modified proteins in mature nodules. These results suggest that sulfenylation may regulate the activity of key proteins involved in nodule development and metabolism.

**Fig. 5.**
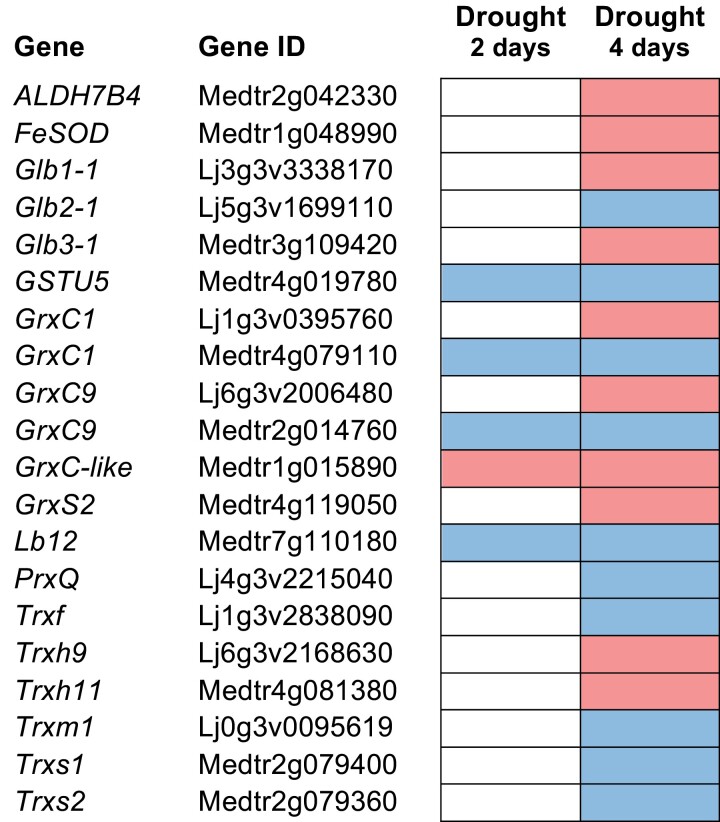
Expression profiles of genes involved in redox homeostasis in nodules of *Lotus japonicus* and *Medicago truncatula* following two or four days of drought stress. Gene up-regulation (>two-fold) and down-regulation (<0.5-fold) are indicated in red and blue, respectively. Expression data were retrieved from LegumeIP v3 (http://plantgrn.noble.org/LegumeIP/gdp/) and Sańko-Sawczenko *et al.,* (2019). Abbreviations: ALDH, aldehyde dehydrogenase; Glb, phytoglobin; Grx, glutaredoxin; GSTU, glutathione transferase tau family; Lb, leghemoglobin; Prx, peroxiredoxin; SOD, superoxide dismutase; Trx, thioredoxin.

Little is known about the possible alterations of protein sulfenylation patterns during the perception and response to abiotic stress. Nevertheless, an important role can be anticipated because Cys oxidation is a highly sensitive and fast way to modify the function of enzymes and transcription factors in response to new conditions. Legumes contain large Trx and Grx families that keep the balance between reduced and oxidized thiols (Alkhalfioui *et al.*, 2008; Tovar-Méndez *et al.*, 2011; Alloing *et al.*, 2018). The analysis of *M. truncatula* transcript profiles showed the up-regulation of several *Trx* and *Grx* genes in plants subjected to salt or drought stress. Interestingly, re-watering of plants for one day after 14 days of drought stress caused a fast increase in the amounts of some *Trx* transcripts both in shoots and roots (Fig. 4). In *L. japonicus*, salt stress induced the expression of two *GrxS* genes in the shoot (Fig. 3; Sanchez *et al.*, 2008). Recently, RNA-sequencing analysis of *M. truncatula* and *L. japonicus* nodules revealed a high number of differentially expressed genes in response to water stress (Sańko-Sawczenko *et al.*, 2019). Transcripts of some Trx*h* and Grx isoforms were induced in both legumes after four days of treatment (Fig. 5). An increase in mitochondrial Trx*o* activity in response to salt treatment was also observed in pea leaves (Martí *et al.,* 2011). These data suggest that restoration and maintenance of protein thiol homeostasis by various Trx and Grx isoforms are important features of the response of legumes to abiotic stress.

### *S*-nitrosylation

The term *S*-nitrosylation is used by the scientific community to refer to the reversible covalent binding of NO to a reactive Cys thiol in a protein (Fig. 2), although some authors prefer to use *S*-nitrosation instead (for further clarification on these terms see Martínez-Ruiz and Lamas, 2004). This PTM has been recognized as a major mechanism by which NO conveys its bioactivity (Lamotte *et al.*, 2014; Gupta *et al.*, 2020). In *A. thaliana* and other species, *S*-nitrosylation of specific proteins is part of the immune and abiotic stress responses (Feechan *et al.*, 2005; Tada *et al.*, 2008; Begara-Morales *et al.*, 2019). One such response is the involvement of NO and *S*-nitrosylation as negative regulators in abscisic acid (ABA) signaling and stomatal closure under physiological and stress conditions. Under drought, ABA accumulates, which enhances the production of NO and the *S*-nitrosylation of the protein kinase OST1/SnRK2.6. This nitrosylation at a specific Cys residue adjacent to the catalytic site blocks the kinase activity of OST1/SnRK2.6, and results in feedback inhibition of ABA signaling in guard cells (Wang *et al.*, 2015). Similarly, diverse studies in legumes have shown that abiotic stress induces changes in the profiles of *S*-nitrosylated proteins. For example, in mitochondria of pea leaves, salt stress caused *S*-nitrosylation of peroxiredoxin IIF (PrxIIF), a key enzyme in ROS- and RNS-mediated redox signaling. Subsequent *in vitro* studies showed that this modification induced a conformational change in the protein that provoked the reduction of its peroxidase activity and the acquisition of a novel function as a *trans*-nitrosylase (Camejo *et al.*, 2013; 2015). In pea leaves, abiotic stress induced changes in *S*-nitrosylation of several enzymes that participate in H_2_O_2_ metabolism, such as catalase (Ortega-Galisteo *et al.*, 2012) and three enzymes of the ascorbate-glutathione cycle (Begara-Morales *et al.*, 2019). Interestingly, this modification increased ascorbate peroxidase activity, but inhibited monodehydroascorbate reductase and dehydroascorbate reductase activities, suggesting a complex crosstalk between NO- and ROS-mediated signaling during the perception and response to abiotic stress.

*S*-nitrosylation was also observed in nodule proteins under optimal growth conditions (Puppo *et al.*, 2013). However, the effect of this modification on protein function, or its role in the response of nodules to stress, is virtually unknown. In one of the few studies addressing these issues, Melo *et al.* (2011) observed that two glutamine synthetase (GS) isoforms of *M. truncatula* are differentially regulated by NO. The activity of plastid MtGS2a was inhibited by *S*-nitrosylation, whereas cytosolic MtGS1a was unaffected by this PTM but inactivated by Tyr nitration (see below). Moreover, in *L. japonicus* and *M. truncatula*, glutathione peroxidases (Gpxs), an enzyme family closely related to Prxs, are *S*-nitrosylated *in vitro* and *in vivo*, which resulted in the partial inhibition of enzyme activities (Matamoros *et al.*, 2015; Castella *et al.*, 2017). These results strongly suggest an important role for NO signaling in the regulation of SNF, although a possible function under stress conditions awaits investigation.

In plant cells, *S*-nitrosoglutathione reductase (GSNOR) and Trxs are involved in *S*-nitrosothiol (SNO) homeostasis (Begara-Morales *et al.*, 2019; Fukudome *et al.*, 2019a; Matamoros *et al.*, 2020). GSNOR is essential for plant development and adaptation to abiotic and biotic stress (Kwon *et al,.* 2012; Lindermayr, 2018; Matamoros *et al.,* 2020). In *A. thaliana* the enzyme is required for acclimation to high temperatures and the null mutants have augmented SNO amounts (Lee *et al.*, 2008). Although a single-copy *GSNOR* gene is predominantly found in most species analysed so far, including *A. thaliana*, many legumes contain two *GSNOR* genes (Matamoros *et al.*, 2020). In *M. truncatula*, comparison of *GSNOR1* and *GSNOR2* expression between control and stress conditions reveals up-regulation of the *GSNOR1* transcript, which encodes the main GSNOR isoform, during the recovery from drought in roots and shoots (Fig. 4). Therefore, the restoration of SNO balance may be a key feature of legume tolerance to drought. Another mechanism for regulation of GSNOR activity is the PTM of the protein. Inhibition of *A. thaliana* GSNOR activity by *S*-nitrosylation led to transient accumulation of SNOs and transmission of the NO signal (Guerra *et al.*, 2016). It was subsequently demonstrated that under hypoxia, besides the inhibition of its enzymatic activity, *S*-nitrosylation induces the selective autophagy of GSNOR due to conformational changes that expose an interaction motif recognized by the autophagy machinery (Zhan *et al.*, 2018). Very recently, PTMs including *S*-glutathionylation, and probably Cys oxidation and persulfidation by H_2_S, were detected in *L. japonicus* GSNOR1 and GSNOR2 recombinant proteins (Matamoros *et al.*, 2020). In addition, GSNOR seems to be central in the crosstalk between Ca^2+^- and NO-mediated signaling under stress conditions. A model was proposed in *A. thaliana* in which salt stress induces the entry of Ca^2+^ into the cell and the formation of the Ca^2+^/calmodulin complex. This interacts directly with GSNOR and inhibits its activity, thereby stimulating SNO accumulation and salt tolerance (Zhou *et al.*, 2016). All these results strongly suggest that plant GSNORs are regulatory hubs that integrate signals mediated by H_2_O_2_, NO, H_2_S, and Ca^2+^, and that they are therefore critical for developmental and stress responses. In addition to GSNOR, mechanisms should exist in plants to keep SNO amounts of proteins under control by denitrosylation. Recently, it has been demonstrated in *A. thaliana* that Trx*h5* catalyses denitrosylation by acting as a selective protein–SNO reductase (Kneeshaw *et al.*, 2014). Interestingly, Trx*h5* (after reduction by NADPH-dependent Trx reductase) decreases excessive protein–SNO amounts, which reinstates signaling by salicylic acid and modulates plant immunity.

Phytoglobins (Glbs; formerly non-symbiotic hemoglobins) are another family of plant proteins that may regulate intracellular NO concentrations, and thereby influence the level of *S*-nitrosylation. Based on their structural characteristics and phylogeny, Glbs are categorized into three classes (for recent reviews see Mira *et al.*, 2016; Becana *et al.*, 2020). Class 1 Glbs display extremely high O_2_ affinities, which makes them unsuitable for O_2_ transport and delivery. They are involved in NO scavenging, probably through their NO dioxygenase (NO + O_2_ → NO_3_^-^) activity. Transgenic *A. thaliana* plants overexpressing *AtGlb1* are more tolerant to hypoxia (Hunt *et al.*, 2002), whereas salt, drought, and cold treatments up-regulate several *Glb* genes in rice (*Oryza sativa*; Shankar *et al.*, 2018). Class 1 Glbs may regulate programmed cell death in certain stress responses (Mira *et al.*, 2016). Plants of *A. thaliana* overexpressing rice *Glb1-2* show increased tolerance to potassium deficiency and lower ROS content, which led the authors to suggest that the corresponding protein might be involved in signaling responses to low nutrients stress (Shankar *et al.*, 2018). However, there is less information on the other two classes of Glbs. Class 2 Glbs have O_2_ affinities similar to those of symbiotic hemoglobins, and the transcript of the single class 2 Glb of *A. thaliana* accumulates in response to cold treatment (Trevaskis *et al.*, 1997). Class 3 Glbs have not been studied in detail in plants. However, their participation in the stress response is likely, based on the NO-related functions of their bacterial homologs (Vieweg *et al.*, 2005).

The functions of Glbs have also been studied in legumes. In *L. japonicus*, the expression of *LjGlb1-1* and the production of NO are concomitantly and transiently induced in roots after 4 h of inoculation with the compatible symbiotic rhizobial species *Mesorhizobium loti*, whereas pathogenic bacteria induced a sustained NO burst triggering the plant’s defense response (Shimoda *et al.*, 2005; Nagata *et al.*, 2008). *LjGlb1-1* is mainly expressed in nodules and strongly induced by hypoxia, NO, and cold stress, whereas another class 1 Glb gene, *LjGlb1-2*, is mainly expressed in leaves and induced by sucrose (Shimoda *et al.*, 2005). Overexpression of *LjGlb1-1* in hairy roots increased nodule number and N_2_ fixing activity, which was attributed to a higher capacity of nodules to scavenge NO and protect nitrogenase (Shimoda *et al.*, 2009). Stable lines overexpressing *LjGlb1-1* also had reduced NO amounts and enhanced N_2_ fixation in mature and senescent nodules (Fukudome *et al.*, 2019a). These lines are more tolerant to flooding, which may be linked to lowered amounts of NO and ROS, compared with the wild-type under these stressful conditions (Fukudome *et al.*, 2019b). In *M. truncatula*, expression of *MtGlb1-1* is also transiently up-regulated after infection with its rhizobial partner, and this induction is thought to decrease NO concentrations in roots, thereby allowing the onset of symbiosis (Berger *et al.*, 2020). These authors also showed that MtGlb1-1 modulates NO concentration throughout nodule development, controlling nodulation and N_2_ fixation. Some Glbs are transcriptionally regulated in response to stress conditions in *L. japonicus* and *M. truncatula*. *LjGlb1-1* expression is down-regulated in response to salinity in the shoot (Fig. 3; Sanchez *et al.*, 2008), but up-regulated in water-stressed nodules (Fig. 5; Sańko-Sawczenko *et al.*, 2019). Also, *MtGlb1-1* expression is down-regulated by drought in roots and shoots, whereas *MtGlb3-1* is induced under salt or drought stress in roots, shoots, and nodules (Li *et al.*, 2009; Sańko-Sawczenko *et al.*, 2019). Remarkably, one *Lb* transcript was induced in the roots of *M. truncatula* in response to salt and re-irrigation following drought (Fig. 4). Further research is warranted to establish the role of Glbs in the stress responses of legumes and, in particular, of the legume-rhizobia symbiosis (for a recent review on this subject, see Larrainzar *et al.*, 2020).

### *S-*glutathionylation

*S*-glutathionylation is the reversible addition of glutathione to a protein via the formation of a disulfide bond with a Cys thiol (Fig. 2). This PTM occurs in response to increases in ROS and NO, and protects thiols from irreversible oxidation. It may also lead to structural and functional changes in the target protein that regulates signal transduction and metabolic pathways (Zaffagnini *et al.*, 2012). Whereas this modification may occur via non-enzymatic mechanisms, deglutathionylation is usually carried out by Grxs. In humans, deregulation of *S*-glutathionylation has been implicated in a number of pathologies, including cancer, cardiovascular disease, and diabetes (Zhang *et al.*, 2018). Much less information is available in plants. Dixon *et al.* (2005) demonstrated that *S*-glutathionylation occurs in response to oxidative conditions in suspension cultures of *A. thaliana*. In the same species, the modification of the catalytic Cys of glycolytic glyceraldehyde-3-phosphate dehydrogenase protects the enzyme from irreversible oxidation. However, the persistence of the glutathionylated state alters protein structure and causes the formation of insoluble aggregates (Zaffagnini *et al.*, 2019). A recent study has shown the effect of *S*-glutathionylation on the protein structure and activity of pea recombinant chloroplastic 2-cysteine Prx (Prx2C) and mitochondrial PrxIIF (Calderón *et al.*, 2017). The modification caused inhibition of the peroxidase activity of the two proteins. However, *S*-glutathionylation provoked specific alterations of the protein structures. It induced the conversion of Prx2C from decamer to dimer, whereas it did not change the oligomerization state of PrxIIF. Interestingly, sulfiredoxin was able to deglutathionylate Prx2C but not PrxIIF, suggesting a role for this protein in the deglutathionylation of specific enzymes. The physiological implications of these discoveries are not clear yet, but it is possible that *S*-glutathionylation of proteins increases under abiotic stresses that perturb the cellular redox state. This PTM may protect the protein from irreversible oxidative deactivation and/or regulate its activity as part of signaling events that control cell metabolism. Grx-mediated deglutathionylation might have a role in the stress response. As discussed above, in *M. truncatula,* several *Grx* transcripts are induced in response to salt or drought conditions (Figs. 4, 5). One of these transcripts (Medtr3g077570) is homologous to poplar (*Populus tremula × tremuloides*) *PtGrxC1*, which encodes a protein that shows deglutathionylating activity *in vitro* (Bedhomme *et al.*, 2012). *S*-glutathionylation might also act as a redox signal during the onset and functioning of the rhizobium-legume symbiosis. This is suggested by the observations that one Grx (SmGrx1) of *S. meliloti* displays deglutathionylation activity and that the corresponding bacterial mutant strain has an impaired symbiotic phenotype (Benyamina *et al.*, 2013). To our knowledge, there is no information about the occurrence of this PTM in nodules.

### Persulfidation

The signaling molecule H_2_S plays an important role in many physiological and pathological processes in plants and animals (Aroca *et al.*, 2018). A mechanism of H_2_S signaling is the conversion of the thiol group (R-SH) of reactive Cys residues into a perthiol (R-SSH, also called a persulfide), in a process known as persulfidation. In solution, H_2_S may exist as deprotonated (H_2_S), monoanion (HS^−^), and dianion (S^2−^) forms. Potential mechanisms for persulfidation include the nucleophilic attack of HS^-^ on oxidized protein thiols such as sulfenic acids, disulfide bridges, and *S*-glutathionylated and *S*-nitrosylated Cys residues (Fig. 2; Mishanina *et al.*, 2015). This PTM may alter protein structure and function because of the decrease in the pK_a_ and the increase in nucleophilicity of the persulfide group (Mishanina *et al.*, 2015). It may also prevent irreversible Cys over-oxidation and preserve protein function (Zivanovic *et al.*, 2019). There is ample evidence that H_2_S mediates abiotic stress responses (reviewed by Hancock, 2019). In *A. thaliana*, a mutation of the H_2_S-producing enzyme l-cysteine desulfhydrase 1 (DES1) leads to drought sensitivity and premature leaf senescence (Jin *et al.*, 2018). Studies with broad bean (*Vicia faba*) indicate that H_2_S functions downstream of H_2_O_2_ in salt stress-induced stomatal movements (Ma *et al.*, 2019), and very recent work with *A. thaliana* has demonstrated the involvement of H_2_S in ABA-induced stomatal closure (Shen *et al.*, 2020). The latter authors proposed the following model: drought causes ABA accumulation that, in turn, induces the expression of *DES1* in guard cells by an unknown mechanism; H_2_S generated by DES1 activity may cause the persulfidation of many downstream proteins, including DES1 itself, thus amplifying the H_2_S-mediated signal; persulfidation of AtRBOHD provokes a ROS burst that triggers stomatal closure; ABA signaling ceases when the high accumulation of ROS triggers the oxidation of persulfides of AtRBOHD and DES1; and oxidized persulfides can be reduced back to thiols by Trxs (Shen *et al.*, 2020).

Information on protein persulfidation and its possible functions in legume physiology is virtually non-existent. A proteomic study is underway in our laboratory with bean nodules at different stages of development, which will provide insights into the possible roles of this PTM in nodule biology. Preliminary results suggest that protein persulfidation is involved in the plant and nodule responses to abiotic stress and in the aging process. This is consistent with interesting results reported recently by Zivanovic *et al.* (2019). These authors used different experimental models, including HeLa cells, *Caenorhabditis elegans*, yeast, and mammals, to show that persulfidation is evolutionarily conserved and plays an integral role in protecting proteins from excessive oxidation. Moreover, they also established a correlation between increased persulfidation, resistance to oxidative stress, and lifespan extension.

### Tyrosine nitration

This PTM consists in the covalent addition of a nitro group (-NO_2_) to one of the two equivalent ortho carbons in the aromatic ring of Tyr residues, to form 3-nitrotyrosine (NO_2_–Tyr; Kolbert *et al.*, 2017). Tyr nitration requires the presence of peroxynitrite (ONOO^-^) or nitrogen dioxide (NO_2_) because NO itself is not reactive enough (Fig. 2). Tyr nitration causes a decrease of the residue pK_a_, enhances its hydrophobicity, and provokes steric restrictions because NO_2_–Tyr is larger than Tyr. In plant cells, Tyr nitration generally leads to loss-of-function of the protein, although there are a few exceptions (Corpas *et al.*, 2013; Kolbert *et al.*, 2017). In pea, this PTM occurs throughout plant development in roots, stems, and leaves, and is increased in aging roots but not in aging leaves (Corpas *et al.*, 2013). The contrasting results between roots and leaves could be related to obvious physiological peculiarities or to differences in the developmental stages of the two organs. Moreover, abiotic stress conditions such as salinity, high light intensity, or low and high temperature increase nitration levels of specific proteins in pea and other plant species (Corpas *et al.*, 2013).

In nodules, Tyr nitration may have an important regulatory role because two key proteins for nodule functioning, GS and Lb, are targets of this PTM. MtGS1a is inactivated by Tyr nitration, and the amount of nitrated protein increases under conditions in which SNF is impaired (Melo *et al.*, 2011). As for Lb, the protein is susceptible to nitration in both the heme and globin. Heme nitration was demonstrated in senescing soybean nodules containing green Lbs. The modified proteins have identical globins to the unaltered red Lbs, but their hemes are nitrated in a vinyl group (Navascués *et al.*, 2012a). In the globin moiety, a Tyr residue located in the distal heme pocket is the major target of nitration (Sainz *et al.*, 2015). Interestingly, the amount of nitrated globin decreased during senescence, suggesting that heme and globin nitration occurs through different mechanisms and/or that globin nitration, but not heme nitration, makes the protein prone to degradation by nodule proteases, as observed with other plant proteins (Castillo *et al.*, 2015).

The significance of protein nitration in redox signaling is still poorly defined. There is evidence that Tyr nitration may interfere with the phosphorylation of the residue and thus regulate signal transduction pathways. Although this stable PTM was categorized as irreversible, recent research in animals has identified denitrase mechanisms that could be also operative in plants (Kolbert *et al.*, 2017).

### Carbonylation and glycation

In cells, metal-catalysed oxidation occurs when free Fe^2+^ or Cu^+^ reacts with H_2_O_2_ and generates hydroxyl radicals (·OH) through the Fenton reaction (Halliwell, 2006). These radicals can irreversibly oxidize amino acid side chains and introduce the carbonyl moiety in proteins (Møller *et al.*, 2011). Carbonyl groups may also be generated indirectly by Michael addition of lipid peroxidation decomposition products to Arg, Cys, histidine, and Lys residues (Møller *et al.*, 2011; Matamoros *et al.*, 2018; Fig. 2). Protein carbonylation contributes to cellular damage caused by stress conditions and age-associated diseases in animals (Höhn *et al.*, 2013). In *A. thaliana* and other plant species, increases in carbonylated proteins were observed in response to abiotic stress (Tanou *et al.*, 2009; Mano *et al.*, 2014). As for legumes, the amount of carbonylated proteins was higher in the leaves of peas grown in the presence of heavy metals (Romero-Puertas *et al.*, 2002). Some of the oxidized proteins were identified as Rubisco and antioxidant enzymes such as glutathione reductase, manganese superoxide dismutase, and catalase. The oxidized proteins were more sensitive to proteolytic degradation. In soybean, protein carbonylation was induced in plants exposed to high CO_2_ and was associated with loss of leaf chlorophyll and reduced photosynthesis (Qiu *et al.*, 2008). Natural senescence (aging) also entails protein carbonylation both in leaves and nodules (Evans *et al.*, 1999; Hernández-Jiménez *et al.*, 2002; Vanacker *et al.*, 2006; Matamoros *et al.*, 2013). It is not clear, however, if irreversible protein carbonylation is only a deleterious consequence of stress conditions or aging or, alternatively, contributes to redox signaling and plant acclimation to stress (Winger *et al.*, 2007; Tanou *et al.*, 2012).

Protein glycation occurs when Arg and Lys residues react with reducing sugars, generating Amadori and Heyns compounds. These glycation products are readily oxidized, yielding relatively stable advanced glycation end products (AGEs). Alternatively, AGEs can be formed by the reaction of Arg and Lys residues with α-dicarbonyls (mainly glyoxal and methylglyoxal), generated by monosaccharide autoxidation under oxidative conditions (Fig. 2). In humans, the cross-link products between proteins and sugars seem to be major contributors to age-related chronic diseases (Höhn *et al.*, 2013). However, very little is known about protein glycation in plants. The *A. thaliana* proteome modified by AGEs, as well as the age-dependent increase of glycation at specific sites, have been reported (Bilova *et al.*, 2017). In this species, osmotic stress also augmented protein glycation (Paudel *et al.*, 2016), and constitutively glycated proteins and age- and drought stress-specific targets have been recently identified (Chaplin *et al.*, 2019). *In vitro* assays showed that glycation inhibited two enzyme activities involved in carbohydrate metabolism, thus demonstrating the impact of this PTM on protein function.

To our knowledge, there is only one large scale study on protein oxidation in legumes (Matamoros *et al.*, 2018). The study revealed that, in bean nodules, carbonylation occurs under normal growth conditions and throughout development, and that this PTM has major effects on two key nodule proteins, malate dehydrogenase and Lb (Fig. 1). Malate dehydrogenase is essential for SNF because malate is the primary source of carbon transported to the bacteroids. Its activity is negatively correlated to the amount of carbonylation. Carbonylation also induces Lb aggregation, probably rendering the protein inactive and more susceptible to degradation by cell proteases. Moreover, numerous glycated proteins have been identified *in vivo*, including 10 plant and 18 bacterial proteins that were age-specifically glycated.

Reactive aldehydes and ketones arising from lipid peroxide degradation are major contributors to protein carbonylation (Mano, 2012; Matamoros *et al.*, 2018). Gpxs and Prxs catalyse the reduction of lipid hydroperoxides and might therefore decrease the rate of carbonylation. Gene expression profiling of *M. truncatula* roots and shoots showed that salt or drought stress up-regulates several *Gpx* and *Prx* genes (Fig. 4; Li *et al*., 2009). It has also been observed that overexpression of reactive carbonyl-scavenging enzymes such as 2-alkenal reductase, alkenal/one oxidoreductase, aldehyde dehydrogenase, aldehyde reductase, and tau class glutathione *S*-transferase confers stress tolerance (Kotchoni *et al.*, 2006; Yin *et al.*, 2010; Yamauchi *et al.*, 2012; Mano *et al.*, 2019). In *L. japonicus*, genes encoding glutathione *S*-transferase, 2-alkenal reductase, and aldehyde dehydrogenase isoforms were up-regulated in response to drought or salt stress (Fig. 3; Sanchez *et al*., 2008; Díaz *et al*., 2010). Similarly, in *M. truncatula*, the transcripts of several enzymes known to scavenge reactive carbonyl species accumulated in response to abiotic stress (Fig. 4; Li *et al*., 2009). Thus, aldehyde dehydrogenase 7B4 (ALDH7B4) plays an important antioxidative role by eliminating surplus aldehydes in plants exposed to high temperatures in combination with drought, wounding, or salinity stress (Zhao *et al.*, 2017). In *M. truncatula,* expression of *MtALDH7B4* is up-regulated in the nodules by drought (Fig. 5) and expression of its homolog in *L. japonicus, LjALDH7B4*, is up-regulated in the shoot by salt stress (Fig. 3). These studies highlight the importance of these enzymes in the defense response of legumes.

On the other hand, in eukaryotic cells the glyoxalase (GLX) system detoxifies glyoxal and methyglyoxal, and thus avoids the accumulation of AGEs. The system involves two consecutive reactions catalysed by GLXI and GLXII. *A. thaliana* contains three *GLXI* and three *GLXII* genes (Schmitz *et al.*, 2017). However, in *M. truncatula* only one *GLXII* gene was up-regulated in response to drought conditions (Fig. 4), suggesting that either the glyoxalase system is not regulated under stress conditions or that it relies on post-transcriptional control for its activation.

## Conclusions and perspectives

In the last few decades, genomic and transcriptomic studies have provided a solid base to understand how plants tolerate and adapt to abiotic stress. As a next step, the thorough analysis of the stress-responsive proteome will allow further elucidation of the metabolic and signaling networks that determine the success of plants to thrive under adverse conditions. Nevertheless, a number of factors that define the proteome, including mRNA alternative splicing, protein subcellular location, protein-protein interactions, and PTMs, add a further degree of complexity. As for PTMs, individual proteins may exist in several modified forms, each with different activity, stability, location, or capacity to interact with other proteins. For a given protein, the same PTM may have different effects depending on the modification site, and a specific PTM can have opposite effects on different proteins (activation or inhibition of enzyme activity). Moreover, several PTMs may coexist in the same protein, resulting in many possible combinations. To further complicate this picture, cellular redox homeostasis is probably regulated independently in each cell compartment, and PTMs may therefore vary with the subcellular location. From a technical viewpoint, redox PTMs are highly dynamic and labile and can be easily altered during sample manipulation if strict protocols are not followed. Although the sensitivity of analytical equipment has been greatly improved over the last few years, low abundant proteins whose modifications could be influential in the plant response, including transcription factors, key regulatory enzymes, and cell membrane receptors, are still generally overlooked.

To date, a large number of proteins are known to be post-translationally modified in response to different stimuli. But in most cases, the effect of these modifications on protein function has not been investigated. To better understand the role of ROS-, RNS- and RSS-mediated PTMs in the stress response, a number of issues need to be addressed. It will be necessary to understand the mechanisms that make PTMs selective toward a specific protein, quantify the percentage of protein molecules that contain a specific PTM, and establish the effect of individual PTMs, or the combination of several of them, on protein function, signaling pathways, and cell metabolism. It will also be required to determine organelle-specific PTMs and identify master proteins whose modification is highly influential. All this complexity often makes it necessary to carry out detailed case study approaches that can be integrated in a general picture of how redox-based protein regulation determines plant stress tolerance. In legumes, a deeper understanding of the molecular and physiological basis of their response to abiotic stress is crucial to improve plant growth and SNF, and thereby to secure food supply with a reduced input of fertilizers.
